# Molecular cloning and expression analysis of peptidase genes in the fish-pathogenic scuticociliate *Miamiensis avidus*

**DOI:** 10.1186/1746-6148-9-10

**Published:** 2013-01-11

**Authors:** Jung Soo Seo, Eun Ji Jeon, Sung Hee Jung, Myoung Ae Park, Jin Woo Kim, Ki Hong Kim, Sung Ho Woo, Eun Hye Lee

**Affiliations:** 1 Pathology Division, National Fisheries Research & Development Institute (NFRDI), 152-1, Haean-Lo, Gijang-Up, Gijang-Gun, Busan 619-705, South Korea; 2 Aquatic Life Disease Control Division, NFRDI, 152-1, Haean-Lo, Gijang-Up, Gijang-Gun, Busan 619-705, South Korea; 3 Department of Aquatic Life Medicine, Pukyong National University, 599-1 Daeyondong, Namgu, Busan 608-737, South Korea; 4 Institute of Fisheries Sciences, Pukyong National University, 295, Dongbaek-ri, Ilgwang-myeon, Gijang-gun, Busan 619-911, South Korea

**Keywords:** Scuticociliates, *Miamiensis avidus*, Peptidases, RT-PCR

## Abstract

**Background:**

Parasite peptidases have been actively studied as vaccine candidates or drug targets for prevention or treatment of parasitic diseases because of their important roles for survival and/or invasion in the host. Like other parasites, the facultative histophagous ciliate *Miamiensis avidus* would possess peptidases that are closely associated with the invasion into the host tissue and survival in the host.

**Results:**

The 17 genes encoding peptidases, including seven cathepsin-like cysteine peptidases, four serine carboxypeptidases, a eukaryotic aspartyl protease family protein, an ATP-dependent metalloprotease FtsH family protein, three leishmanolysin family proteins and a peptidase family M49 protein were identified from a *Miamiensis avidus* cDNA library by BLAST X search. Expression of genes encoding two cysteine peptidases, three leishmanolysin-like peptidases and a peptidase family M49 protein was up-regulated in the cell-fed ciliates compared to the starved ciliates. Especially, one cysteine peptidase (MaPro 4) and one leishmanolysin-like peptidase (MaPro 14) were transcribed more than 100-folds in the cell-fed ciliates.

**Conclusions:**

The genetic information and transcriptional characteristics of the peptidases in the present results would be helpful to elucidate the role of peptidases in the invasion of scuticociliates into their hosts.

## Background

Parasite peptidases have been widely studied as potential vaccine candidates or promising targets of anti-parasitic agents for prevention or treatment of parasitic diseases, because of their crucial roles in completing the life cycles or diseases they produce. In many protozoan parasites that cause malaria, trypanosomiasis, leishmaniasis, amebiasis, toxoplasmosis, giardiasis, cryptosporidiosis, and trichomoniasis, the major roles of parasite peptidases include invasion by degradation of host cells and tissues, degradation of mediators of the immune responses, and the catabolism of host proteins for parasite growth and survival [1-5].

In the facultative histophagous *Miamiensis avidus* (*=* synonym of *Philasterides dicentrarchi*), which causes high mortality in cultured olive flounder (*Paralichthys olivaceus*) in Korea [6,7]*,* peptidases might play important roles in the process of transforming of the ciliates from the free-living form into the invasive, infectious form, which might make the peptidases as candidates for vaccine antigen or treatment drug target. It has been reported that peptidases secreted by *Philasterides dicentrarchi* can degrade type-I collagen, modulate host cellular immune responses, and induce apoptosis of leucocytes [8-11]. Moreover, *P. dicentrarchi* peptidases could affect host humoral immune responses by degrading the host immunoglobulins and reducing host complement activity in fish serum and ascitic fluid [12].

Although there are several reports about the important roles of peptidases in scuticociliate *M. avidus*, no studies combined with genetic identification of peptidase genes and gene expression related to the function on the invasion have been performed. Therefore, the purpose of this study was to identify peptidase genes that are expected to have features related to infection of *M. avidus* by comparison of expression level between the cell-fed and the starved ciliates.

## Methods

### Ciliates

Ciliates were isolated from ascitic fluid of an infected olive flounder *Paralichthys olivaceus* collected from a local fish farm in Korea, and were identified as *Miamiensis avidus* using species-specific oligonucleotide primers [6]. Chinook salmon embryo (CHSE)-214 cells, incubated at 20°C in Eagle’s minimum essential medium (MEM, Sigma, St. Louis, Mo, USA) supplemented with 10% heat-inactivated fetal bovine serum (FBS), were used as grazing material to grow the ciliates under axenic culture conditions.

To obtain cell-free cultured ciliates, ciliates harvested from routine CHSE cell-feeding cultures were transferred to filtered sea water without any nutrient components and starved at 20°C for at least 1 month. To obtain cell-fed ciliates, ciliates were inoculated in sufficiently grown CHSE-214 cells in routine MEM supplemented with 10% heat-inactivated FBS or in sufficiently grown CHSE-214 cells in filtered seawater supplemented with 10% heat-inactivated FBS or were intraperitoneally injected into olive flounder. The ciliates from different culture conditions were harvested using a method described previously [13]. Briefly, the ciliates were harvested by centrifugation at 200 × *g* for 5 min, and washed more than 3 times by centrifugation at 150 × *g* for 5 min in Hanks’ balanced salt solution (Sigma) or filtered seawater. The experiments using fish and treatment of dead fish were performed in accordance with the guideline approved by Ministry for Food, Agriculture, Forestry and Fisheries.

### RNA preparation, cDNA library construction and expressed sequence tag (EST) analysis

Total RNA from CHSE-cultured *M. avidus* was prepared using Trizol reagent (Invitrogen, Carlsbad, CA, USA) according to the manufacturer’s instructions. Poly A+ RNA from the total RNA prepared from CHSE-cultured *M. avidus* was isolated using the Stratagene Absolutely mRNA Purification Kit (Stratagene, La Jolla, CA, USA). A cDNA library was constructed using the ZAP Express cDNA Synthesis Kit and Gigapack III Gold packing extract (Stratagene) according to the manufacturer’s instructions. The titer of constructed cDNA library was 5.6 × 10^5^ plaque-forming units (pfu)/ml.

The expressed sequence tags (ESTs) were analyzed by DNA sequencing of kanamycin resistant *Escherichia coli* clones containing cDNA fraction-harbored phagemid (pBK-CMV) after mass excision of the lambda phage library. DNA sequencing was conducted with T3/T7 phagemid sequencing primers using an ABI3730 automatic sequencer (96-capillary, Applied Biosystems, Foster City, CA, USA) and Applied Biosystems BigDye® Terminator Cycle Sequencing Kits v3.1, in accordance with the manufacturer’s recommendations. A total of 1,265 EST sequences, obtained cDNA library of *M. avidus* RNA, were analyzed by sequence comparison with previously reported sequences in the EMBL/GenBank databases using the BLAST X search program of the National Center for Biotechnology Information (NCBI). The domain search of deduced amino acid sequences was analyzed using the SMART web and the NCBI protein blast program.

### Real-time reverse transcriptase polymerase chain reaction (RT-PCR) of peptidase genes in *Miamiensis avidus*

RT-PCR was performed to further verify the expression patterns of the isolated peptidase genes. Total RNA was isolated from cell-free cultured ciliates and cell-fed ciliates using the RNeasy Mini Kit (Qiagen, Valencia, CA, USA). Total RNA was isolated several times, and the pooled total RNA was used for cDNA synthesis. cDNA was synthesized from 1 μg of total RNA using Superscript II Reverse Transcriptase and Oligo (dT) 20 primer (Invitrogen). RT-PCR was performed using Fast Start SYBR Green Master Mix (Roche, Indianapolis, IN-, USA) and 100 ng of synthesized cDNA in a 20 μl reaction volume. Quantitative PCR was conducted using an iQ5 Multicolor Real-Time PCR instrument (Bio-Rad, Hercules, CA, USA), and the β-tubulin (BTU) gene was used as the internal control for normalization. Thermal cycling conditions were one cycle of 3 min at 95°C (initial denaturation) followed by 40 cycles of 10 s at 95°C, 10 s at 55°C, 20 s at 72°C. The specific PCR primers for amplification of *M. avidus* peptidase genes were designed from the unique sequences obtained by analysis of ESTs using the OLIGO 5.0 software (National Bioscience) (Table 1) and the expected sizes of PCR products are listed in Table 2. The results of RT-PCR from triplicate experiments were expressed as mean Ct (Cycle threshold) values and standard deviation. The fold change in relative gene expression under the different culture conditions was determined by the 2^−ΔΔCt^ method [14,15]. ΔΔCt (delta delta Ct) values were calculated using an equation, where ΔΔCt = (Ct_MaPro_ - Ct_BTU_)_cell-fed_ - (Ct_MaPro_ - Ct_BTU_)_cell-free_. Significant differences of Ct values were determined by Paired t-test after normalization using those of β-tubulin gene.

**Table 1:** **Characterization of the peptidases in *Miamiensis avidus***

**Name of clone**	**Number of clone**	**Protein length (amino acids)**	**Matched proteins (Species, Accession number)**	**Homology (E-value)**	**Putative domains contained**
MaPro 1	3	355	Cathepsin L-like cysteine protease (*Uronema marinum,* AAX51228)	31% (1x10^-57^)	signal sequence, I29, peptidase C1
MaPro 2	2	346	Cathepsin L-like cysteine protease (*Uronema marinum,* AAX51228)	36% (3x10^-68^)	signal sequence, I29, peptidase C1
MaPro 3	2	355	Cathepsin L-like cysteine protease (*Uronema marinum,* AAX51228)	36% (2x10^-54^)	I29, peptidase C1
MaPro 4	1	342	Cathepsin L-like cysteine protease (*Uronema marinum,* AAX51228)	37% (1x10^-57^)	signal sequence, I29, peptidase C1
MaPro 5	1	342	Papain family cysteine protease containing protein (*Tetrahymena thermophila* SB210, EAR82238)	49% (2x10^-59^)	signal sequence, I29, peptidase C1
MaPro 6	2	337	Cathepsin B (*Uronema marinum*, AAR19103)	59% (9x10^-146^)	signal sequence, propeptide, peptidase C1
MaPro 7	1	362 (partial)	Cathepsin C (*Danio rerio*, AAH64286)	37% (1x10^-57^)	peptidase C1
MaPro 8	2	479	Serine carboxypeptidase (*Oxytricha trifallax*, AMCR01008778)	30% (1x10^-57^)	signal peptide, peptidase S10
MaPro 9	1	518	Serine carboxypeptidase family protein (*Tetrahymena thermophile*, XP_001031619)	36% (3x10^-73^)	signal peptide, peptidase S10
MaPro 10	5	473	Serine carboxypeptidase S28 family protein (*Tetrahymena thermophile*, XP_001013945)	53% (1x10^-155^)	signal peptide, peptidase S28
MaPro 11	1	477	Serine carboxypeptidase 24-like isoform 1 (*Glycine max*, XP_003519151)	32% (9x10^-62^)	signal peptide, peptidase S10
MaPro 12	1	381	Eukaryotic aspartyl protease family protein (*Tetrahymena thermophile* XP_001016313)	48% (2x10^-107^)	Asp domain
MaPro 13	1	283 (partial)	ATP-dependent metalloprotease FtsH family protein (*Tetrahymena thermophila* SB210, EAR83289)	79% (8x10^-159^)	AAA domain, Peptidase M41
MaPro 14	4	731	Leishmanolysin family protein, putative (*Ichthyophthirius multifiliis*, EGR31368)	34% (1x10^-82^)	Signal peptide, Peptidase M8, EGF-like domain, Furine-like repeat
MaPro 15	5	1102	Leishmanolysin family protein, putative (*Ichthyophthirius multifiliis*, EGR31368)	33% (1x10^-73^)	Signal peptide, Peptidase M8, EGF-like domain, Furine-like repeat
MaPro 16	1	693	Leishmanolysin family protein, putative (*Ichthyophthirius multifiliis*, EGR33997)	29% (1x10^-64^)	Signal peptide, Peptidase M8, EGF-like domain, Furine-like repeat
MaPro 17	1	708	Peptidase family M49 (*Tetrahymena thermophila* SB210, EAR87978)	61% (0)	Peptidase M49

**Table 2:** List of primers used in quantitative reverse-transcription PCR (RT-PCR)

**Target clone/gene**	**Primers**	**Sequences (5**^**′**^**- 3**^**′**^**)**	**Product size (bp)**
MaPro 1	Forward	TGCTTCCACTTCAGTTTTATCAGTCG	266
	Reverse	GGTTAAGTTCAACTGTGGGGATTTCTAA	
MaPro 2	Forward	TCTTGAGAGCTTCTGCTGCCAC	271
	Reverse	TCTTGGATGTTTAATTCGGTGCTGT	
MaPro 3	Forward	AATCCAACGAAGACATCAGAATCTTCT	246
	Reverse	CAGGGACTTATCTGGAAGGTCTGGA	
MaPro 4	Forward	TAGCTTCAATTGCTTCTGGTAGTCTTG	277
	Reverse	ATCCATGTTTATTCCACATAGTCCATTAC	
MaPro 5	Forward	ATTTCAAGCGATTGGAAGCTAAGAATC	308
	Reverse	AATAATCCCAAATAGAATATTACCCATCTTC	
MaPro 6	Forward	ACTTGGACTGCTGGATACAACAAAC	310
	Reverse	CGGTGGAGATTCTGGTTTGAAC	
MaPro 7	Forward	CGGTGGAGATTCTGGTTTGAAC	212
	Reverse	TTCGGCATCAACAGAATGGTAGATAC	
MaPro 8	Forward	ACGTTTTATTAGAAAGCCAAGGTAACC	260
	Reverse	GGTATTTTCGTCGGTGTATTTGTAGCT	
MaPro 9	Forward	GGGAAAAGGAAACTCTGCATTCG	348
	Reverse	CATCCATTTTCAGCAGTGTACAGTTCTAT	
MaPro 10	Forward	CAGATAATGGCTCTACTAATATTGCACTC	243
	Reverse	AGGGATCTTCACTTCCATTTGTGAATAC	
MaPro 11	Forward	ATTTGGCTCAATGGAGGACCTG	217
	Reverse	CAGCGGTATTATCATCAGTGTAAGAGT	
MaPro 12	Forward	CCACCTACAAACCCCAAGGAGAC	268
	Reverse	GCGAAAGAGTTGTCTTCCCAGACT	
MaPro 13	Forward	CAGCTCTTTTAACAGAAGGAGCTACAC	352
	Reverse	TTTCTGAACTTCGGTATCCACCATA	
MaPro 14	Forward	AGGGTATCTTCGAACAGCTCTTCG	319
	Reverse	CATTGGGACAAGAGACTGAACAGTC	
MaPro 15	Forward	AGCCTTGGAATGGAAATACTTTCGCTG	319
	Reverse	CCAACACAGTATCCGTTAGAGCTACAG	
MaPro 16	Forward	ACTCACGGATAGAACAATGCTCTTGC	314
	Reverse	TTAAAGTGCTTGCGAGCCACTTCC	
MaPro 17	Forward	TTGCAAGTTTCCTCTGGATTTGAATC	327
	Reverse	TCCGTATATAAGTTCAATTGTGGCATC	
β-tubulin	Forward	GTATGATCATTGATAACGAAGCCCTCTACG	323
	Reverse	TCTGGGATCGGCGGCGCACATCATG	

## Results

### Isolation and sequence analysis of peptidase genes from the *Miamiensis avidus* cDNA library

We isolated 32 clones harboring peptidase gene sequences from 1,265 EST clones of the *M. avidus* cDNA library and obtained 17 different peptidase gene sequences including 15 full open reading frames and two partial sequences after DNA sequencing with the T3 and T7 primers and gene specific primers. These 17 genes encoded peptidase proteins including seven cysteine peptidases, four serine carboxypeptidases, a eukaryotic aspartyl protease family protein, an ATP-dependent metalloprotease FtsH family protein, three leishmanolysin family proteins and a peptidase family M49 protein, respectively. The characteristics of the peptidase proteins, including frequency, protein length, homology to previously deposited proteins and conserved domains are shown in Table 1. The conserved domains were analyzed using the SMART web program, Genbank BLAST program, and other literature [16-22].

**Figure 1:**
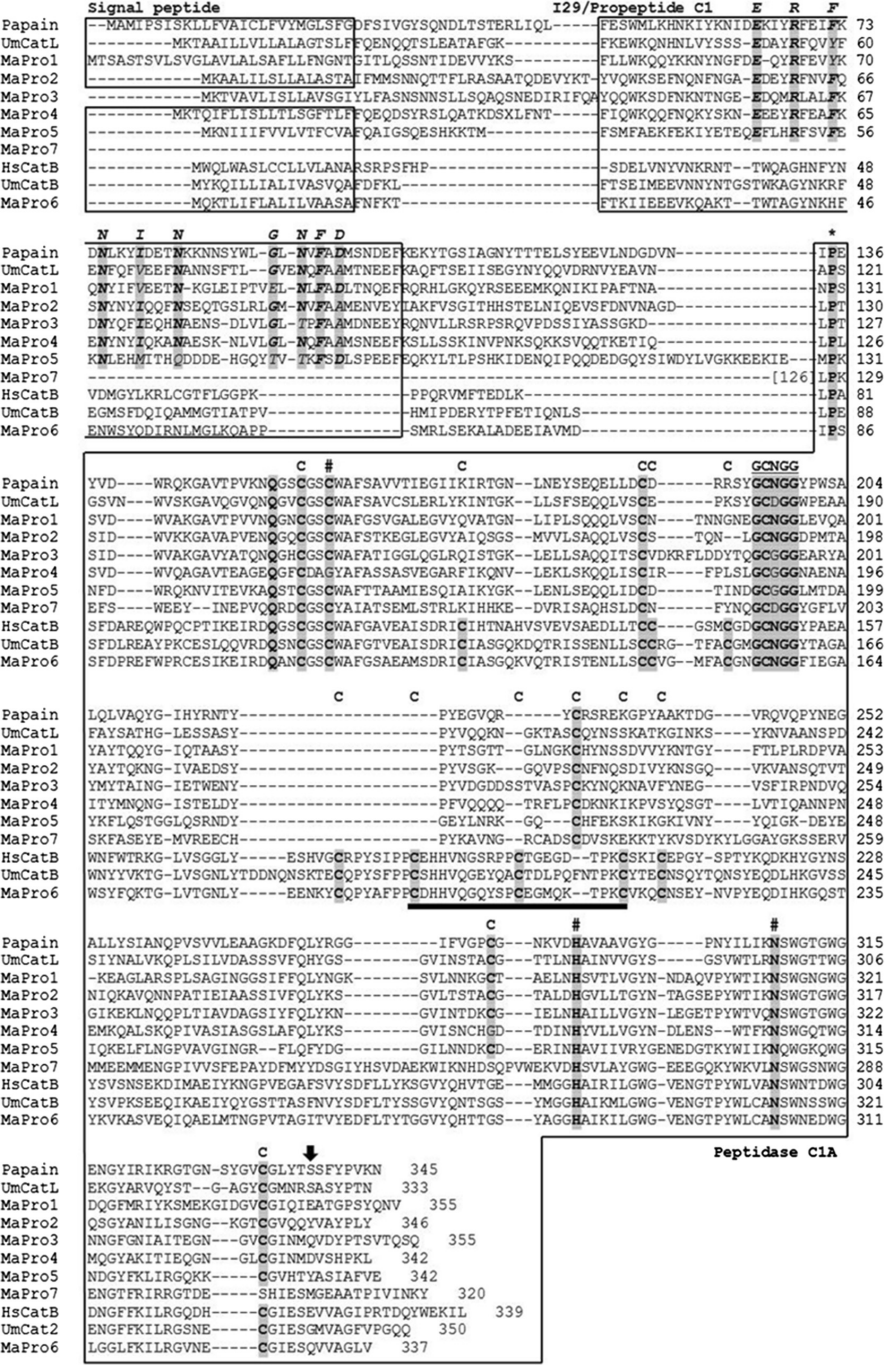
**Multiple alignment of the deduced amino acid sequences of *Miamiensis avidus *cysteine peptidases.** The deduced amino acid sequences of *Miamiensis avidus* cysteine peptidases MaPro 1 to - MaPro 7 were aligned with Papain (the type peptidase of C1A superfamily, GenBank: AAB02650), *Uronema marinum* cathepsin L-like protein (UmCatL, GenBank: AAX51228), *Homo sapiens* cathepsin B protein (HsCatB, GenBank: AAH10240) and *Uronema marinum* cathepsin B protein (UmCatB, GenBank: AAR19103). The signal peptide, I29 (Inhibitor 29)/ Propeptide, Peptidase C1A domains are boxed. Conserved signatures of cathepsin L family proteins (ERFNIN and GNFD) with slight modifications in I-29 peptide of the cathepsin L-like proteins (MaPro 1- MaPro 5) are highlighted in bold, italic, grey shaded and indicated by ERFNIN and GNFD above the alignment. The catalytic triad residues (C, H and N) are marked in bold, grey shaded and indicated by sharp (#). Conserved proline residues at position 2 of the mature proteins are indicated by asterisk (*) and cysteine residues forming disulphide bonds are in bold, grey shaded and indicated by C above the alignment. S_2_ subsite determining enzyme substrate specificity is indicated by a black vertical arrow above the alignment. The predicted ‘occluding loop’, which is the specific feature of cathepsin B-like peptidases, is presented in only MaPro 6 and is indicated with a black thick underline.

**Figure 2:**
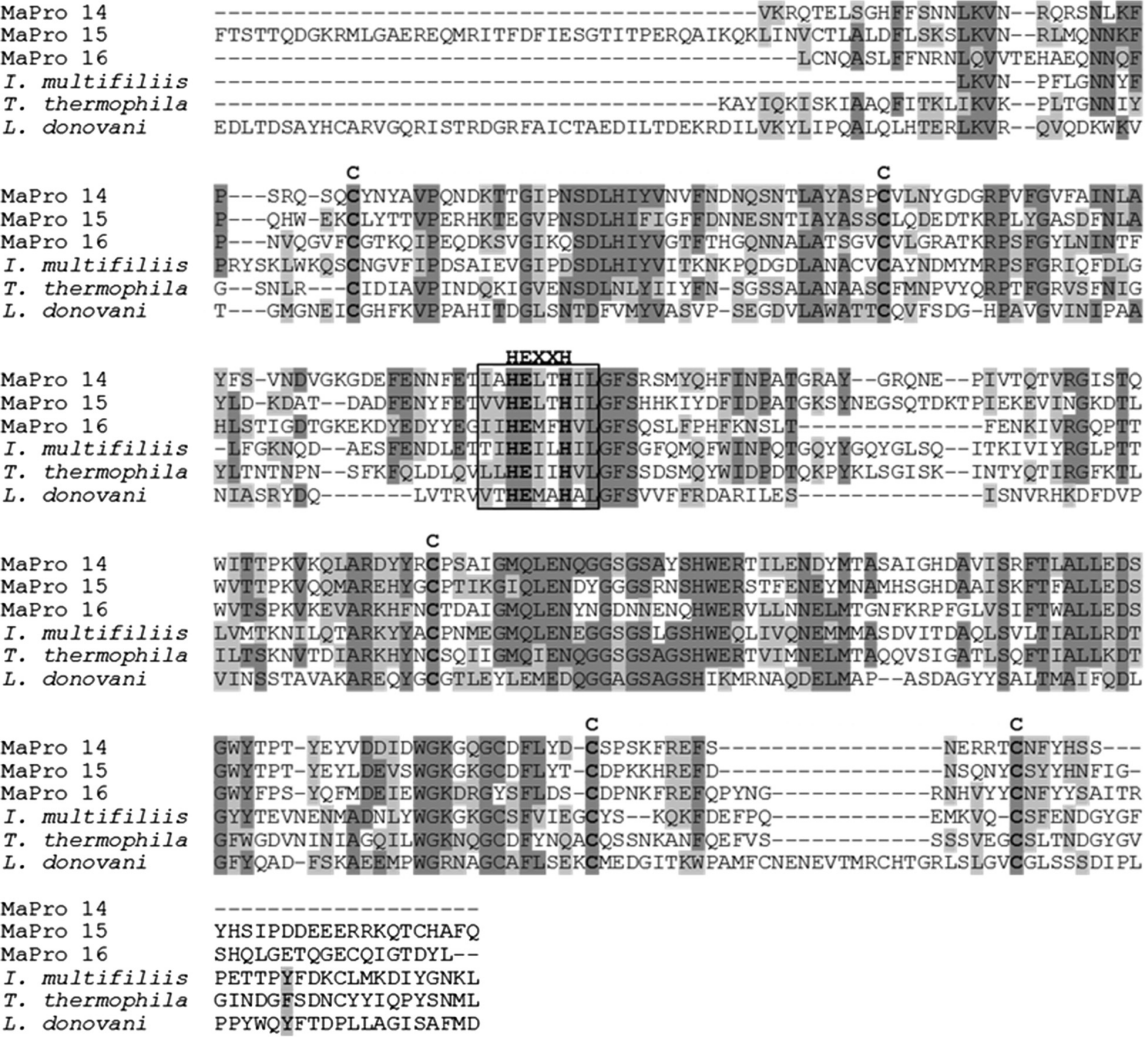
**Multiple sequence alignment of peptidase M8 domains of the *Miamiensis avidus *leishmanolysin family proteins.** The peptidase M8 domains of *M. avidus* leishmanolysin family proteins MaPro 14 – MaPro 16 were aligned with the previously deposited protozoan sequences in protein databases. The zinc-binding signature, HEXXH motif conserved in many kinds of metallopeptidases is shown in bold and in square box, and cysteine residues are presented in bold. The GenBank accession numbers of aligned leishmanolysin family proteins of *Ichthyophtirius multifiliis, Leishmania donovani and Tetrahymena thermophila* are EGR30431, XP_001024633 and AAA29244, respectively.

The seven cysteine peptidases (MaPro 1 - MaPro 7) commonly contained the peptidase C1A domain in their mature protein sequences, which contained catalytic triad residues (C, H, and N) essential for peptidolytic activity, glutamine (Q) residue involved in the formation of the oxyanion hole, the structurally important GCNGG motif, six cysteine residues forming three disulfide bonds and S_2_ subsite determining enzyme substrate specificity (Figure 1). However, in MaPro 4, both the first amino acid residue of the catalytic triad and the fifth cysteine residues forming a disulfide bond were G, not C. The five cysteine peptidase proteins had signal peptide sequences, except for MaPro 3 and MaPro 7 which had no detectable signal peptide and obtained partial sequence containing only the peptidase C1A domain, respectively. The conserved signatures of cathepsin L family protein, ERFNIN and GNFD motifs with slight variation were presented in the type I29 inhibitor domain of MaPro 1 - MaPro 5 proteins presented in other cathepsin L proteins. ERFNIN motif seems to function as an autoinhibitory domain [23] and an important role of GNFD motif in processing and folding of C1A proteses have been shown using site-directed mutagenesis studies [24]. Sequence identities of MaPro 1 – MaPro 5 with the cathepsin L protein of *Uronema marinum* (UmCatL, AAX51228), which is one of the most related scuticociliate species, were 30.7%, 35.9%, 35.6%, 37% and 22.4%, respectively. The MaPro 6 protein sequence contained a signal peptide, a propeptide C1 domain, and a peptidase C1A domain containing twelve conserved cysteine residues forming disulfide bonds and the “occluding loop” which is a specific feature of cathepsin B-like peptidases distinct from other C1 superfamily peptidases and had a 58.9% identity to the cathepsin B-like peptidase protein of *U. marinum* (Figure 1). We identified four different serine carboxypeptidase proteins and three different leishmanolysin family proteins in *M. avidus*. All four serine carboxypeptidase proteins contained a signal peptide, and three had a peptidase S10 domain, and one had a peptidase S28 domain that commonly exist in serine carboxypeptidase proteins. The leishmanolysin family proteins had a signal peptide, a peptidase M8 domain, and a different number of epidermal growth factor (EGF)-like domains and furine-like repeats. As the individual lengths of the proteins were varied (Table 1), common peptidase M8 domains were aligned for comparative purposes with other reported leishmanolysin family protein sequences of similar protozoa (Figure 2). As shown in Figure 2, the HEXXH motif and cysteine residues conserved in other metallopeptidase proteins were present [22].

### Real-time reverse transcriptase polymerase chain reaction (RT-PCR) of peptidase genes

The relative level of peptidase genes expression was analyzed by quantitative RT-PCR using cDNA prepared from *M. avidus* RNA grown under the different culture conditions. As shown in Table 3, the MaPro 4, MaPro 7, MaPro 14, and MaPro 17 genes, which corresponded with a cathepsin L-like cysteine peptidase, a cathepsin C-like cysteine peptidase, a leishmanolysin-like peptidase and a peptidase family M49 protein gene, showed more than 2-fold increased expression in the cell-fed ciliates compared with that in the starved ciliates. Two leishmanolysin-like peptidases, MaPro 15 and MaPro 16, showed detectable Ct values only in the cell-fed ciliates during our RT-PCR process (Table 3).

**Table 3:** **Real time RT-PCR analysis of peptidase genes in *Miamiensis avidus *under the different culture conditions**

**Name of clone**	**Mean Ct**^**a **^**value (SD**^**b**^**)**		**Fold increase in expression (2**^**−ΔΔCt**^**(SD))**	
	**Cell-free culture**	**Cell-feeding culture**	**Cell-free culture**	**Cell-feeding culture**
MaPro 1	28.48(0.51)	22.67(0.32)	1.05(0.40)	1.01(0.22)
MaPro 2^**^	25.38(0.24)	21.25(0.12)	1.01(0.16)	0.31(0.03)
MaPro 3	27.43(0.34)	22.11(0.10)	1.02(0.23)	0.71(0.05)
MaPro 4^**^	34.96(0.28)	21.95(0.24)	1.02(0.20)	147.58(24.89)
MaPro 5	33.19(0.44)	29.71(0.63)	1.03(0.30)	0.21(0.08)
MaPro 6^**^	23.93(0.36)	20.01(0.19)	1.02(0.27)	0.27(0.04)
MaPro 7^*^	33.61(0.31)	25.72(0.11)	1.01(0.22)	4.19(0.33)
MaPro 8^**^	26.94(0.21)	24.11(0.31)	1.01(0.14)	0.13(0.03)
MaPro 9^*^	29.33(0.58)	26.79(0.58)	1.05(0.42)	0.11(0.04)
MaPro 10	32.67(1.44)	26.44(0.29)	1.29(0.86)	1.34(0.27)
MaPro 11	30.26(1.30)	25.09(0.25)	1.24(0.79)	0.64(0.11)
MaPro 12^**^	25.57(0.08)	22.90(0.29)	1.00(0.06)	0.11(0.02)
MaPro 13^*^	34.37(0.34)	31.15(1.30)	1.02(0.22)	0.21(0.16)
MaPro 14^**^	32.51(0.41)	19.37(0.01)	1.03(0.27)	159.78(1.40)
MaPro 15	N/A^c^	31.12(0.76)		
MaPro 16	N/A	31.52(0.57)		
MaPro 17^*^	34.68(0.77)	26.87(0.32)	1.10(0.54)	4.04(0.84)
β-tubulin	23.37(0.45)	17.55(0.22)		

^a^Ct: Cycle threshold, ^b^SD: standard deviation, ^c^N/A: No detectable Ct value was obtained within 40 cycles, ^*^ : Significant differences P<0.05, ^**^ : Significant differences P<0.005.

## Discussion

Scuticociliatosis, causing severe mortality in South Korea, has been considered one of the most serious fish diseases than any other countries [6,7]. Although several reports have revealed that some chemotherapeutics showed effective scuticocidal activities *in vitro*[25-27], finding effective chemotherapeutics is still remained to be difficult *in vivo*. Therefore, efficient vaccine development is urgently to prevent diseases. Several studied have attempted to develop vaccines using whole cells and have shown some positive results by obtaining good protection against scuticociliate infection following vaccination [28-30]. However, as there are some limitations for in vitro mass and economic culture of scuticociliates for commercial use, subunit vaccines should be developed with development of cost-effective methods of antigens. Selecting suitable target antigens that can induce effective protective responses may be the most important factor to develop effective subunit vaccines. Among scuticociliate antigens, the crucial roles of peptidases in infection of host fish have been already reported by previous studies [8-12,31].

In the present study, *M. avidus* were starved for at least 1 month by inoculation in the filtered seawater without any additional nutrients and cells. Similar to what occurs during the free-living stage, these starved ciliates may lose their ability to destroy fish tissue due to a reduction in protease activities they need. Quantitative RT-PCR was performed to investigate mRNA expression of the identified peptidase genes in relation with the cell-feeding parasitic stage of *M. avidus*. The results showed that two cysteine peptidases, a leishmanolysin-like peptidase and a peptidase family M49 protein genes were up-regulated more than 2-fold in the cell-fed ciliates than in the starved ciliates. Among them, one cysteine peptidase gene (MaPro 4) and one leishmanolysin-like peptidase gene (MaPro 14) showed 100-fold higher expression in the cell-fed ciliates. Expression of two other leishmanolysin-like peptidase genes (MaPro 15 and MaPro 16) was detected only in the cell-fed ciliates.

The differential expression of the cysteine peptidases in the cell-fed ciliates might be an important part of *M. avidus* pathogenesis as shown in previous studies [8-12]. Many parasite cysteine peptidases and their important roles in pathogenesis have been well documented in a variety of parasites and inhibitors of cysteine peptidases have been developed for anti-parasitic chemotherapy, as cysteine peptidase inhibitors can selectively inhibit parasite peptidases without untoward toxicity to the host [32-36]. In this study, we cloned seven different cysteine peptidases genes and two of them (MaPro 4 and MaPro 7) were differentially up-regulated in the cell-fed ciliates. We obtained the mRNA sequences of three leishmanolysin-like family proteins containing the metalloprotease M8 domain and found differential mRNA expression in the cell-fed ciliates. Leishmanolysin, which is also known as the gp63 protein, is a metalloprotease found in protozoan parasites including *Leishmania* and *Trypanosoma*. This protein is the most abundant cell surface protein during the promastigote stage of the parasite and is attached to the membrane by a glycosylphosphatidylinositol anchor [37-40]. The protective effects of gp63 immunization have been recently demonstrated using various vaccine formulations by many researchers [41,42]. In fish pathogenic haemoflagellates *Cryptobia* spp., metallopeptidase activity was only found in the pathogenic strain of *C. salmositica*, its activity decreased significantly with long-term *in vitro* culture, and the purified metallopeptidases could lyse fish red blood cells [43-45]. Moreover, metallopeptidase activities could be neutralized by either a monoclonal antibody or a natural anti-peptidase or the antibody against the DNA vaccines [46-48].

Recent technological advances in whole genome sequence analyses and comparative genomic analyses have revealed that there are more than 90 peptidase homologs in a single organism such as *Plasmodium falciparum, Tetrahymena thermophila* and *Ichthyophthirius multifiliis*[49-51]. In protozoa, 254, 578, 480 and 95 peptidase genes have been identified in *Ichthyophthrius multifiliis, Paramecium tetraurelia, Tetrahymena thermophile,* and *Plasmodium falciparum*, respectively [51]. Like other parasites, *M. avidus* may express many peptidase proteins to undergo various biological processes including parasite survival and pathogenesis. In this study, we obtained 17 different peptidase genes from a *M. avidus* cDNA library by ESTs sequence screening, and the results of differential mRNA expression related to pathogenesis were also obtained. Based on the analysis of structurally conserved regions and motifs presented in the deduced amino acid sequences of each peptidase proteins, five cathepsin L-like cysteine peptidases, one cathepsin B-like cysteine peptidase, one cathepsin C-like cysteine peptidase, four serine carboxypeptidase, a eukaryotic aspartyl protease family protein, an ATP-dependent metalloprotease FtsH family protein, three leishmanolysin family proteins, and a peptidase family M49 protein were identified although there were some structurally differences with previously reported similar proteins. Although the number of identified peptidase genes obtained from this study was relatively lower than expected, this is the first report of cloning and mRNA expression of peptidase gene homologs as important virulence factors in *M. avidus*. Moreover, the information of exact protein sequence obtained from this study could help to perform futher studies to develop specific inhibitors.

We are currently performing studies on the actual activities using recombinant proteins of cloned peptidase genes to understand whether these proteins are biologically active at the protein level. We will further analyze of *M. avidus* genome using large scale-genome analysis techniques to identify more peptidase sequences, and will perform combined research of transcriptional analysis and enzymatic activities of each peptidase proteins.

## Conclusions

In conclusion, the genetic information obtained from this study could help to design specific vaccine formulations and inhibitors of peptidases to prevent and control of fish scuticociliatosis caused by *M. avidus,* although further studies to elucidate the exact roles of these peptidases should be conducted.

## Competing interests

The authors declare that they have no competing interests.

## Authors’ contributions

EHL contributed to the design of the study, performing experiments, data analysis and preparation of the manuscript. JSS contributed to the design of the study and advised on data analysis and review of the manuscript. SHW and EJJ participated in data collection and experimental procedure. SHJ, MAP, JWK, KHK involved with the review of the manuscript. All authors read and approved the final manuscript.
